# Exploring How Children and Young People With Sickle Cell Disease and Their Families Want to be Involved in Research: A Qualitative Study

**DOI:** 10.1111/hex.70242

**Published:** 2025-03-28

**Authors:** Jane Chudleigh, Addassa Follett, Ethan Mcfarlane‐Griffith, Derick Abuo, Josh, Imani, Pru Holder

**Affiliations:** ^1^ Cicely Saunders Institute, King's College London London UK; ^2^ Sickle Cell Society London UK; ^3^ Children and Young People, Co‐creators of the Animation Birmingham London UK; ^4^ Cicely Saunders Institute King's College London London UK

**Keywords:** children and young people, patient and public involvement and engagement, sickle cell anaemia, sickle cell disease

## Abstract

**Background:**

There is growing emphasis on the importance of Patient and Public Involvement and Engagement in research to ensure it addresses the needs of the target population. Disparities exist in terms of underserved and underrepresented groups, including children, young people and minority ethnic groups.

**Objective:**

This study sought to listen, hear and understand what is important for children and young people with sickle cell disorder and their families in terms of research involvement and co‐produce resource(s) to enable inclusive and equitable research involvement.

**Design:**

A sequential qualitative study consisting of three work packages to (i) identify structures and processes, (ii) identify resources and (iii) co‐produce an animation to enable equitable and inclusive research involvement for children and young people with sickle cell disorder and their families.

**Results:**

Children and young people with sickle cell disorder, their parents and researchers highlighted several important considerations to ensure inclusive and equitable research involvement, including practical elements, the age and stage of development of the child, as well as condition‐specific issues such as stigma. The use of a variety of approaches and techniques is vital to support inclusive, equitable and sustained involvement and engagement in research activities.

**Conclusion:**

There are many potential barriers that need to be overcome to involve children and young people with sickle cell disorder and their families in research. These include the need for flexible timings of activities, clear expectation setting, consideration of group dynamics and the impact of different ages and stages of development of the children and young people involved, and ensuring appropriate recognition and compensation for their time. Listening, hearing and understanding what is important to children and young people with long‐term conditions and using a variety of approaches is vital to support inclusive, equitable and sustained involvement and engagement in research.

**Patient and Public Contribution:**

Patients (children and young people with sickle cell disorder), caregivers and people with lived experience were involved in conducting the study, analysis and interpretation of the data and preparation of the manuscript.

**Trial Registration:**

NCT06293222.

## 1 Introduction

There is growing emphasis on the importance of Patient and Public Involvement and Engagement (PPIE) in research to ensure it addresses the needs of the target population [1]. However, to date, this has predominantly focused on adult populations, with significantly less momentum in involving children and young people (CYP) [2]. CYP have limited opportunities for research involvement [3, 4]. There is also a lack of evidence on the best models for research partnerships that can effectively include CYP [5]. However, it is vital to include the views of CYP to improve their understanding of how illness may affect their lives and gain a greater understanding of childhood conditions [6]. However, challenges to their involvement include their potential vulnerability, issues of capacity, legal protections, and the possible need to adapt study designs and outcome measures for different age groups. Careful consideration should also be given to the associated risks and benefits of involving CYP in research [6].

Addressing issues related to health equity [7] and accessibility [8] necessitates a diverse and inclusive approach to PPIE. Research should be accessible to those who would benefit the most [6]. However, current evidence indicates that research participants are typically from a narrow demographic [9]—older adults, white ethnic groups and higher socio‐economic backgrounds. This leaves many groups underserved and underrepresented, including children, young people, minority ethnic groups, socio‐economically disadvantaged groups, migrants, asylum seekers and individuals with mental health or multiple health conditions.

While the term ‘sickle cell disease’ is mainly used in the literature, participants in our study expressed a preference for the term ‘sickle cell disorder (SCD)’; therefore, this term has been used throughout this study. SCD predominantly affects individuals of African, Mediterranean, Arabian and Indian origins [10]. Disparities that exist in terms of accessing and receiving healthcare for racial/ethnic groups have been directly related to poor outcomes associated with SCD [11, 12]. A recent report highlighted substantial evidence of racial inequalities for those with SCD in the United Kingdom [13]. Another found a pattern dating back over many years, of sub‐standard care, stigmatisation and lack of prioritisation for patients with SCD in the United Kingdom. This resulted in patients with SCD losing trust in and feeling scared to access healthcare systems, expecting poor treatment from those supposed to care for them and fearing it is only a matter of time until they encounter serious care failings [14]. Factors perceived to contribute to sub‐standard treatment included lack of effective joined‐up care and community care being viewed as inadequate or non‐existent leading to unnecessary hospital admissions [14]. This emphasises the need to ensure people with SCD are involved in designing and prioritising research that directly affects them. However, distrust in health professionals, lack of awareness and understanding regarding research eligibility, financial barriers and illness beliefs have been cited as reasons for non‐engagement in research by people with SCD; this has not changed over time [14, 15].

Shifting the balance of power towards wider participation, empowerment, diversity and equality is crucial. This approach would enable movement beyond minimal consultation towards meaningful partnerships and collaborations with a broader range of people and communities [16]. Co‐production with CYP and their families is considered an important approach to ensure research remains relevant to them and minimises risk [6].

The purpose of this study was to listen, hear and understand what is important for CYP with SCD and their families in terms of research involvement and use this information to co‐produce resource(s) to enable inclusive and equitable PPIE with them.

## 2 Materials and Methods

### 2.1 Design

**Figure 1:**
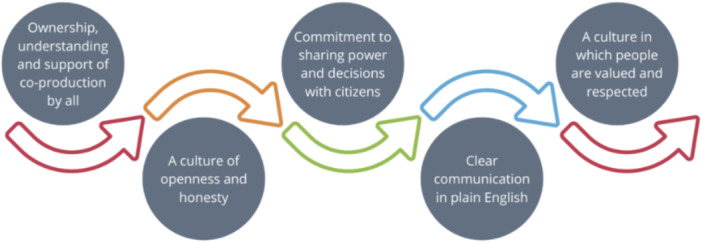
Co‐production model [17].

This qualitative study was underpinned by the co‐production model developed by NHS England and the Coalition for Personalised Care (see Figure 1) [17]. This model acknowledges that people with lived experience of a condition are best placed to advise on the support and services required to meet their needs.

This model was deemed appropriate since it advocates the sharing of power and an ethos of valuing and respecting contributions. This was considered particularly important in engaging the CYP in the co‐production work.

### 2.2 Ethics and Consent

Ethical and research governance approval for this study was obtained from King's College London Research Ethics Committee Reference: LRM‐23/24‐38222. Written informed consent (and assent for children) was obtained from all subjects involved in the study.

### 2.3 Recruitment

In collaboration with the Sickle Cell Society, we purposefully recruited two groups of participants:

Group A: (i) CYP (aged 10–18 years) with sickle cell disorder and (ii) their parents.

Group B: Researchers from the Faculty of Nursing, Midwifery and Palliative Care at King's College London.

### 2.4 Study Design

We used a sequential, qualitative study design involving three work packages, each consisting of two workshops (six in total). The workshops were audio‐recorded and transcribed verbatim.

#### 2.4.1 Work Package 1

We held separate CYP and adult online scoping workshops (*n* = 2) using Padlet software, a virtual notice board that can be used for collaborative online working, with Group A(i) CYP with SCD and A(ii) their parents recruited via the Sickle Cell Society to:

(i) Explain what research is, how it is conducted, relevant policies and the purposes of the work being undertaken.
(ii) Identify structures and processes for equitable and inclusive PPIE for CYP with SCD and their families.
(iii) Identify relevant and appropriate training/education/skills needed to enable CYP with SCD and their families to engage in research.

The CYP workshop was facilitated by two researchers (J.C. and P.H.) to assist with activities and engage participants.

#### 2.4.2 Work Package 2

Two online workshops were held, one with parents and researchers and one with parents and children. These enabled each to identify resources needed for meaningful PPIE with CYP with SCD and their families to take forward into Work Package 3. The first workshop reviewed work undertaken during the scoping workshops in Work Package 1 and considered how to best work with CYP during the second workshop. The second workshop was facilitated by two researchers (J.C. and P.H.) and an artist from Nifty Fox Creative to assist with activities to engage participants. The purpose of the workshops was to consider what a resource that would enable researchers to effectively work with CYP with SCD in the future might look like.

#### 2.4.3 Work Package 3

This consisted of two online workshops with CYP with SCD and their parents facilitated by two researchers (J.C. and P.H.). During these workshops, participants worked on and refined the design of the final output, including co‐producing the script and voiceover of the final version of the animation.

#### 2.4.4 Celebration Event

An online meeting with children and parents during which certificates were presented, and the final resource was viewed.

### 2.5 Data Analysis

**Figure 2:**
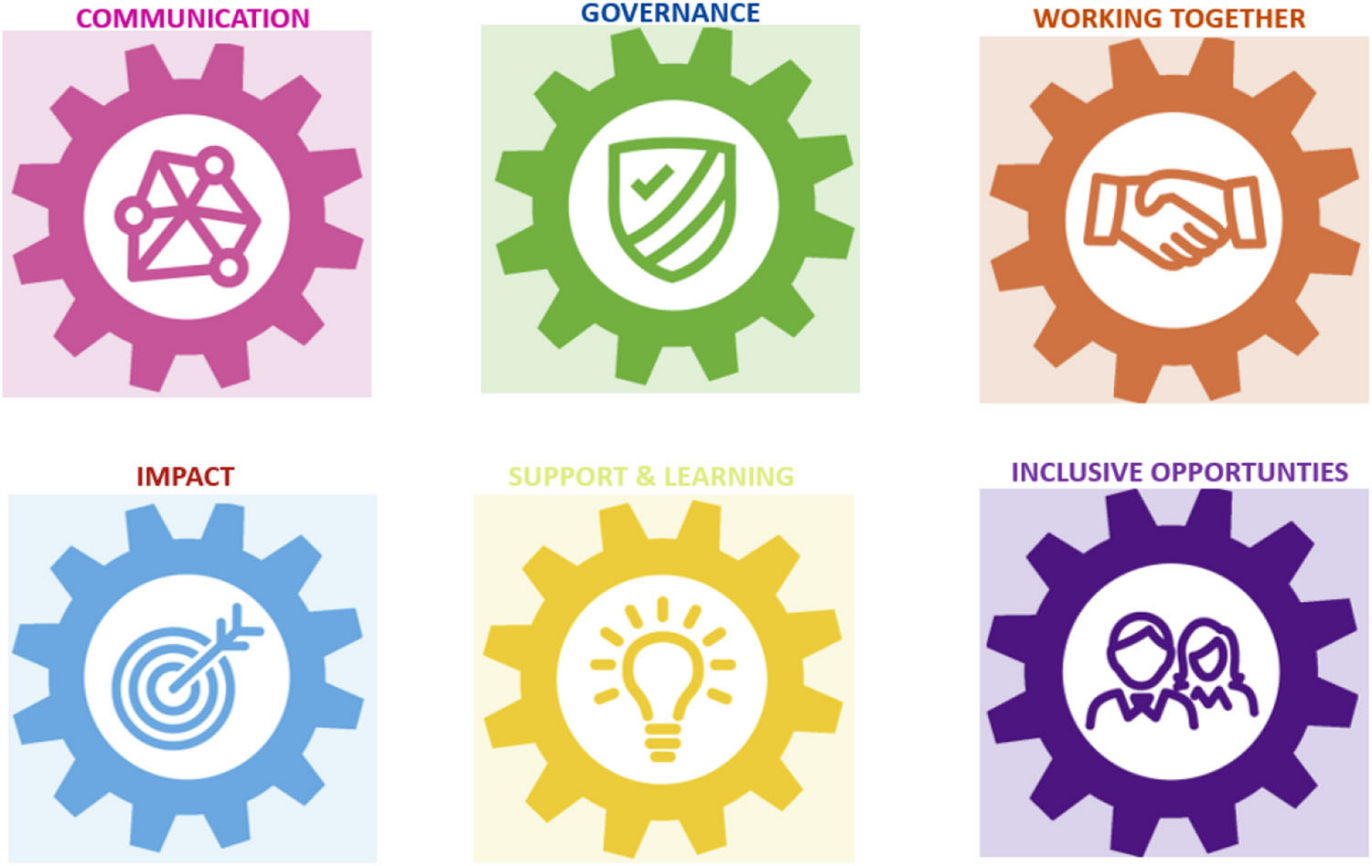
UK Standards for Public Involvement [19].

The five stages of Framework analysis [18] were employed by two members of the research team (J.C. and P.H.) to analyse data collected during Work Package 1. The UK Standards for Public Involvement [19] (Figure 2) were used as the analytical framework. These consist of six standards: inclusive opportunities, working together, support and learning, communications, impact and governance. In the first stage, J.C. and P.H. familiarised themselves with the data by reading the workshop transcripts. In Stage 2, key themes in the transcripts were compared with the a priori selected UK Standards of Public Involvement [19] framework (Figure 2). During Stage 3, data from transcriptions were coded, and relevant participant quotes were identified and indexed. In Stage 4, codes were charted and summarised. In the final stage, a summary of the main descriptive comments was developed to enable interpretation of the data.

Transcribed data from workshops in Work Packages 2 and 3 were subject to content analysis [20]. This enabled views expressed to be summarised and developed into broad statements about CYP's preferences about research involvement whilst also enabling representation of the depth and complexity conveyed within the data. This enabled the drafting of the transcript to accompany the animation.

### 2.6 PPIE

We worked with the Lead Mentor at the Sickle Cell Society (A.F.) to gain feedback on the design and progress of the project. We also worked with CYP with SCD (E.M.‐G., D.A., J. and I.) to co‐write the paper reporting the findings of the project.

## 3 Results

### 3.1 Study Participants

The total study sample was *n* = 18 and consisted of seven CYP (five boys and two girls) with SCD aged 10–17 years [participant group A(i)], seven mothers and one father of children with SCD [participant group A(ii)] all of whom were from ethnic minority backgrounds and three researchers (participant group B) one of whom was White British and two of whom were from ethnic minority backgrounds, see Table 1.

**Table 1:** Study Sample^1^.

CYP	Age (Years)	Gender	Parent
Faye	10	Female	Mother, Isabelle
Edward	10	Male	Mother, Maria
David	11	Male	Mother, Gabrielle
James	11	Male	Mother, Josie
Mark	12	Male	Mother, Sarah
Imogen	12	Female	Mother, Sadie
Elias	17	Male	Father, Ben
—	—	—	Mother, Olive

Researchers	—	Gender	—
Mary	—	Female	—
Fran	—	Female	—
Karen	—	Female	—

^1^ Pseudonyms have been used.

### 3.2 Work Package 1

Seven parents attended the first workshop, and six CYP attended the second workshop. The UK Standards for Public Involvement [19] was used as the framework for analysis and data are presented using the six standards as themes.

#### 3.2.1 Inclusive Opportunities

The timing and location of research activities were felt to be important. CYP suggested that to enable them to be involved, it would be important to offer options to accommodate out‐of‐school activities and acknowledge that due to their condition, they may be tired in the evenings and in the mornings at weekends.

‘…I would prefer it around the afternoon or like the evening, just not the morning.’

Imogen, aged 12 years

Parents felt it important that researchers ensure opportunities for younger children to be involved in research. While they appreciated that it might be easier to engage with older children, they felt listening to the needs of younger children was important as these may differ.

‘…it would be good to hear what the younger children have to say…a lot of the research that is offered is usually 16 and up, so it's good to be involved in something that is with children at least 10. You still have a voice, still want to be heard.’

Maria (Mother)

Parents also thought it would be important to involve siblings of children with long‐term health conditions. While they recognised that siblings were not always directly impacted by the condition, that is, SCD, they felt all members of the family could be indirectly impacted, and this was not always acknowledged.

‘…There was a time that my son was telling me that I give too much attention to my daughter. I have to explain to him that it's because she's mostly unwell. So that's the reason why I gave her much attention because she needs me more than him. So, I think that would be great for them to know.’

Josie (Mother)

‘…I think it's important for children with sickle cell trait to also be involved…with my younger son. Oh, we have to go to hospital again. Oh, I can't do this…I do feel that mummy guilt because we do have to change things.’

Maria (Mother)

CYP and parents stated that they would prefer to undertake activities with other children online rather than face‐to‐face. Children viewed online activities as a better use of their time and felt it negated the need to use their energy travelling. However, they also felt that everyone should be given an option of how they would like to be involved, whether online or face‐to‐face. Whichever option was used, parents stated that research activities should be carefully planned to make them engaging.

‘…make it like fun and dynamic for children.’

Josie (Mother)

‘…make it fun and interesting, like maybe monopolize on the social media 'cause we're in a digital world, so make things that can sort of probably have link to TikTok or something. That sort of what the children are using these days.’

Sadie (Mother)

#### 3.2.2 Working Together

CYP, in the current study, stated they were happy to work with researchers to improve care for CYP with SCD. However, they did not wish to undertake any preparatory work as they likened this to having to do homework for school.

‘…I'm quite happy to find out everything when I come on to here because it surprises me.’

James, aged 11 years

Parents thought their children would enjoy working with other children, both those older and younger than themselves, but felt careful consideration needed to be given to how this worked in practice. Some parents felt that working with older children was beneficial as they viewed them as role models from whom they could learn.

‘…benefit her being older because sometimes they might be a bit scared to start talking and then she starts talking. So then it's like, you know, like a Big Brother or sister. They'll like kind of follow suit. So I think it has its advantages…’

Sadie (Mother)

‘…when my son goes to the support groups, he will sometimes look for the older, especially the older boys as in like did this happen to you or have you been through this?’

Maria (Mother)

However, they also acknowledged that sometimes it might be appropriate to split the groups so that children of similar ages worked together to keep the groups ‘safe’.

CYP felt it was important to think of them as co‐creators of services and felt it vital that researchers think about what the children might want to discuss as well as simply focusing on the research question. Parents also stated that they would like to work with health professionals when undertaking research so the people looking after their children could hear what was important to them.

‘…Health professionals, particularly because they also deal with…they are the one that handle them, when there's a problem or when they have crisis. So I think healthcare practitioners too should be involved very much involved.’

Gabrielle (Mother)

#### 3.2.3 Support and Learning

CYP and parents in the current study stated that they felt it important that in return for being involved in research, they would like to learn more about their condition.

‘…I think they think he [son] knows a lot more than he's supposed to, but I just feel it's important that he knows more about it [SCD] ‘cause, I don't have sickle *cell*, I have the trait. He has sickle cell, but also something around how they relate that to their peers.’

Maria (Mother)

‘…to learn more about sickle cell, any information…I like someone explaining it to me…I would still want something [information] to keep.’

Imogen, aged 12 years

‘…you should explain what sickle cell does to the body and how it works… it's better when it's explained by a professional.’

James, aged 11 years

CYP also suggested that research activities could be carefully designed so they fit in with extracurricular activities they might be undertaking, such as brownies/cubs/scouts and/or the Duke of Edinburgh Award. CYP also stated that they would like to learn more about how research is conducted and learn research skills, for example, by being involved in interviewing other children or healthcare professionals. Also, learning more about presentation and writing skills. CYP spoke about how this could enhance their own knowledge and skills, which could be negatively impacted by missing school due to their condition. Parents also felt that learning more about SCD could increase their children's confidence.

‘…you need to like boost the children's confidence…addressing this stigma in this disease will also help in boosting the children's confidence, not letting them feel that they are not like every other child, or that they are not fit enough, or that they are not strong enough. So because this may affect the child's self‐esteem and confidence as well, so boosting the child's confidence will help.’

Gabrielle (Mother)

#### 3.2.4 Communication

Parents in the current study felt it was vital that the purpose of any research was made explicitly clear from the outset; they wanted to know if the research was focused on their children as individuals and their well‐being or if it was simply to find out more about their condition.

‘…what's the aim of the research, this information you need to gather what are you using them for, is it to improve our well‐being or is it just to gather information to talk about sickle cell disease?’

Gabrielle (Mother)

‘…what is it that we're going to gain out of the research? Is it to help improve services? Is it for another reason? Is it both?’

Sadie (Mother)

CYP and parents in the current study also stated that they should be free to choose a pseudonym if they wished but would be happy to share their ages. Parents also stated that the research environment should be a safe space where their children could freely express themselves without fear of reprisal.

‘…if they've got their own ideas, they may be different from your explanation and your intention.’

Sadie (Mother)

#### 3.2.5 Impact

CYP and parents stated that they wanted involvement to be recognised and suggested that receiving a certificate for CYP would be a good way of doing this. They also suggested an online ceremony at the end of a research project to celebrate their success and involvement in the project.

‘…about the certificates, it might be nice to like, on the back of that do like a little, I don't know, virtual online ceremony at the end where everyone gets presented…like make it a bigger thing than it is. So, it's like something to look forward to when you finish like you complete it, this is what you can get.’

Sadie (Mother)

‘…the certificates…ones that are like, say we're involved and what we've done to earn it.’

Imogen, aged 12 years

Parents also stated they wanted research findings to be made widely available so that they could be used to raise awareness of their condition and reduce any stigma or misconceptions.

‘…raising awareness because especially in our community, people don't talk about it. They don't discuss about sick sickle cell. They think it's a taboo.’

Josie (Mother)

#### 3.2.6 Governance

Parents and children spoke about the importance of setting expectations at the start of any research activities. It was felt that these should be developed in partnership with CYP and their families, so they maintained ownership of them. They also felt that it should be made clear from the outset what would happen if these expectations were not adhered to.

‘…not rules. But you know, like I said, of expectations of what to expect or what will be covered or what you kind of need the dos and don'ts sort of thing maybe…and consequences if things aren't followed.’

Sadie (Mother)

Participants felt it was vital that anyone involved in research, but particularly CYP were compensated for their time as this made them feel valued. It was also felt that the process for being compensated in terms of rewards and incentives that would be available following participation in any research activities should be transparent from the outset.

‘…rewards and incentives is a big thing for children now. They're going to get something out of it. If you're thinking about especially my daughter's 12. So that preteen stage that attention span, it's very sort of you've got to grab them, and you've got to give them something that they're going to want. So, I think whatever that may be that you can afford to give, whether it's vouchers, I don't know. But I think incentivize your sort of research projects where you can.’

Sadie (Mother)

CYP stated that they were used to engaging in online activities through school, particularly since the Covid‐19 pandemic, but felt that there was an expectation that they should have their cameras on. However, CYP in the current study stated that this should be optional if CYP with a health condition were involved in research since they may not feel well enough or be self‐conscious about their appearance if they were unwell.

‘…If I'm not well, could have it [camera] off.’

Elias, aged 17 years

### 3.3 Work Package 2

**Figure 3:**
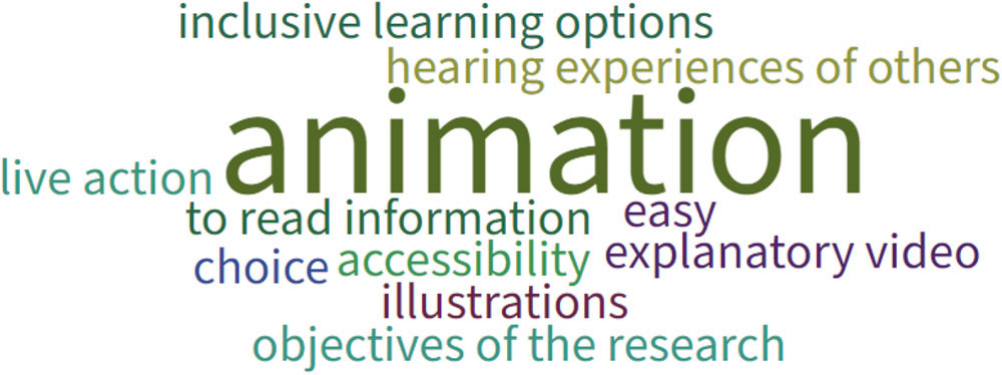
Information or resources if considering being involved in research.

**Figure 4:**
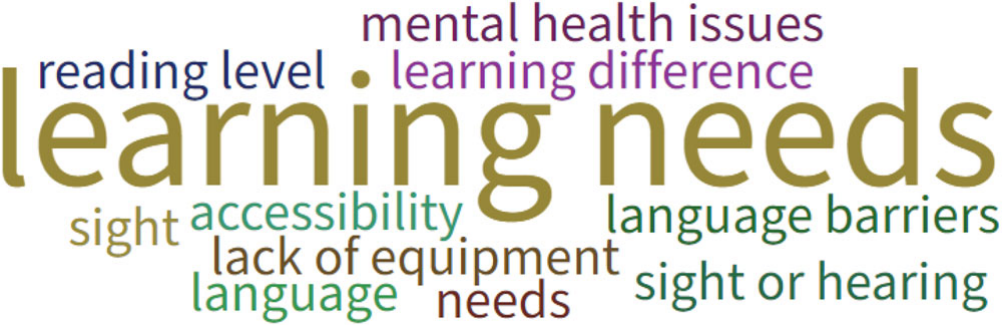
Reasons why someone might need different or extra information or resources to be involved in research.

**Figure 5:**
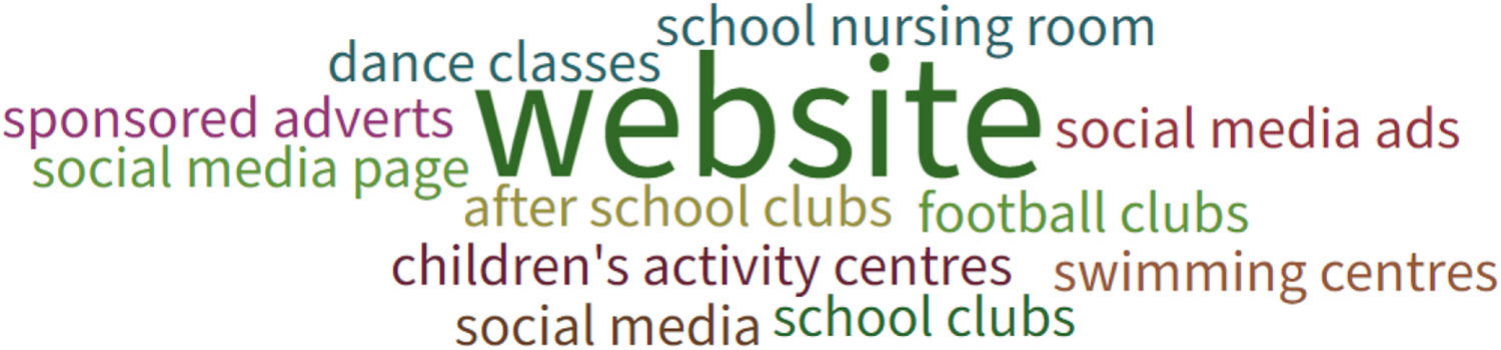
Hosting information and resources for CYP.

During the first workshop, Poll Everywhere, an online tool that enables users to create and collect responses using a range of activities, was used to gather feedback from participants. Primarily this focused on exploring with parents (*n* = 6) and researchers (*n* = 3) how to work with children to co‐produce resource(s) to enable inclusive and equitable PPIE with them. Parents and researchers were asked (i) what information or resources they would want or need if they were considering being involved in research, (ii) any reasons why someone might need different or extra information or resources if they wanted to be involved in research and (iii) where information or resources should be hosted to enable CYP to be involved in research. Results can be seen in Figures 3, 4, 5, respectively. Parents and researchers favoured an animation to inform potential participants about being involved in research (Figure 3) that could be hosted on a website (Figure 5). They also considered it important to develop strategies to overcome potential barriers to inclusive involvement and engagement in research, such as learning differences, language barriers and disabilities (Figure 4).

**Figure 6:**
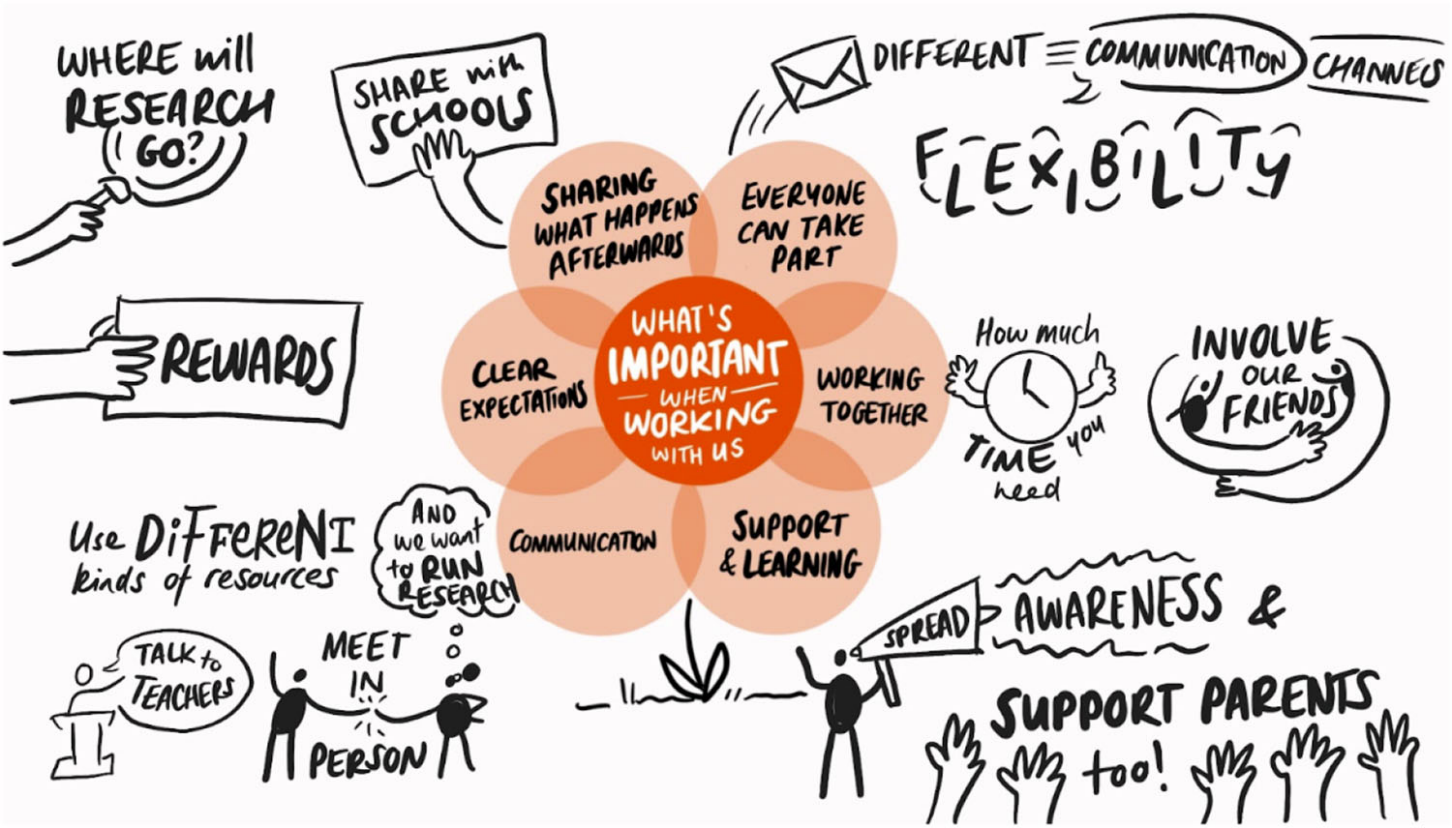
What is important when working together.

A free digital whiteboard application that permits real‐time collaborative work among research participants, Google Jamboard (GJ), was used with CYP with SCD (*n* = 5), their parents (*n* = 7) and researchers (*n* = 3) during the second workshop in this Work Package. This enabled exploration of how they could use their ideas from Work Package 1 to co‐produce a resource for inclusive and equitable PPIE in research with CYP with SCD and their families. CYP with SCD, their parents and researchers were asked what is important to them when working together for research purposes. A summary of responses can be seen in Figure 6.

Following this, CYP with SCD, their parents and researchers were asked how their ideas about conducting research with CYP with SCD should be shared to ensure it is accessible to those who would benefit from it the most. Suggested choices included an animation, illustrations, infographics, a website or something else. CYP with SCD, their parents and researchers were asked to vote, using green dots they could move around the screen, for their preferred option.

CYP with SCD, their parents and researchers selected an animation and discussed how their ideas could be shared with a variety of stakeholders who may be interested in undertaking research with CYP with SCD and their families. CYP also chose the name of their group, ‘Sickle Cell Superheroes’, to spearhead the animation.

### 3.4 Work Package 3

**Figure 7:**
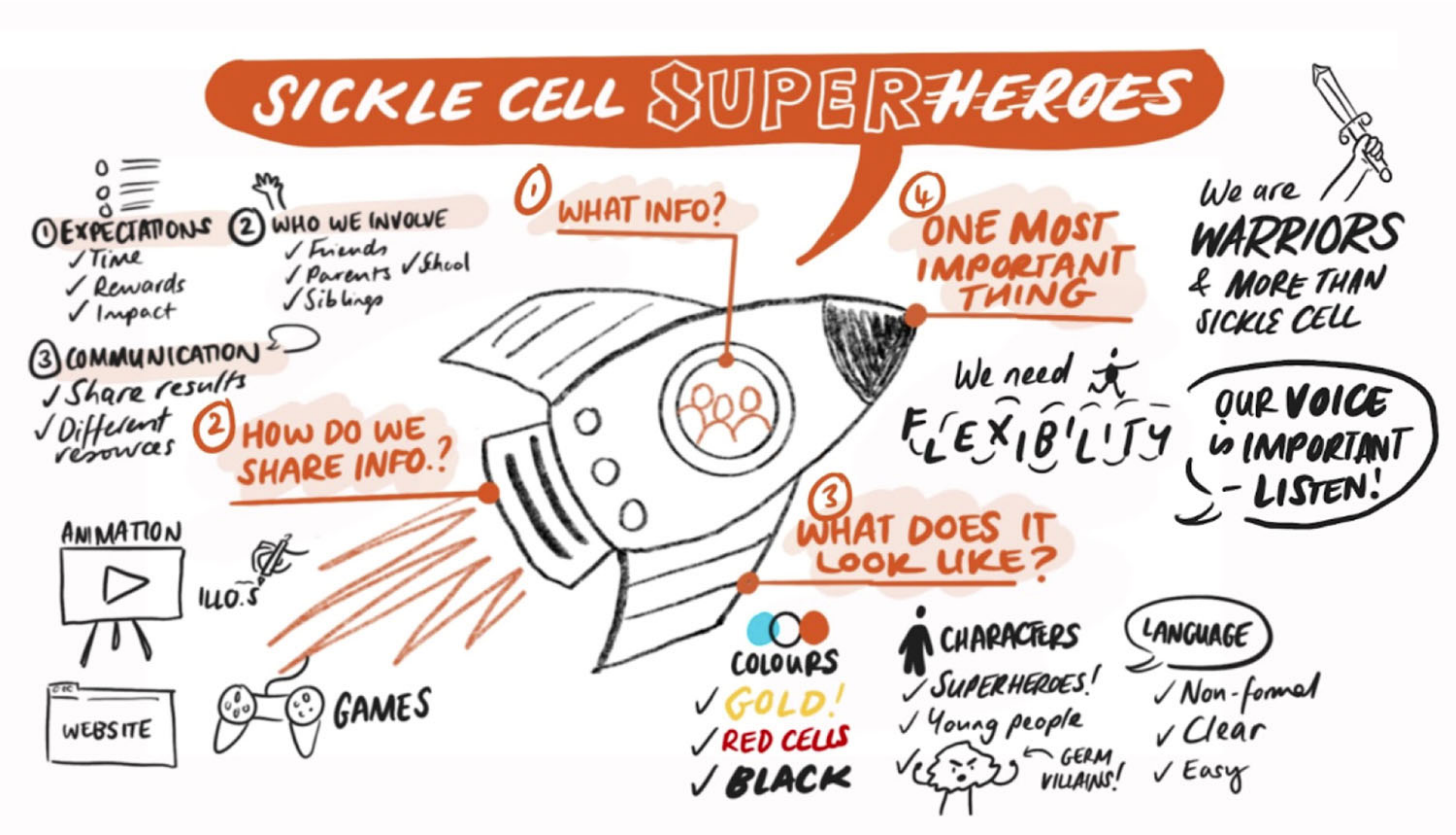
CYP with SCD, their parents and researchers' ideas for the animation.

CYP with SCD (*n* = 6), their parents (*n* = 7) and researchers (*n* = 2) were asked to consider the characters, colours, language and letter styles that they would like to see used for the animation they had chosen in Work Package 2. CYP indicated they would like the characters in the animation to be children and have red clothes as a reminder of the importance of red blood cells. They stated that the characters should resemble people who are most likely to be affected by SCD. They also stated that the language should be informal and should be targeted at CYP and not adults. They were also asked the most important thing they would want researchers to remember from their animation. The CYP were particularly keen that the overall message should be that SCD is only a part of them and does not define who they are. A summary of their ideas is shown in Figure 7.

**Figure 8:**
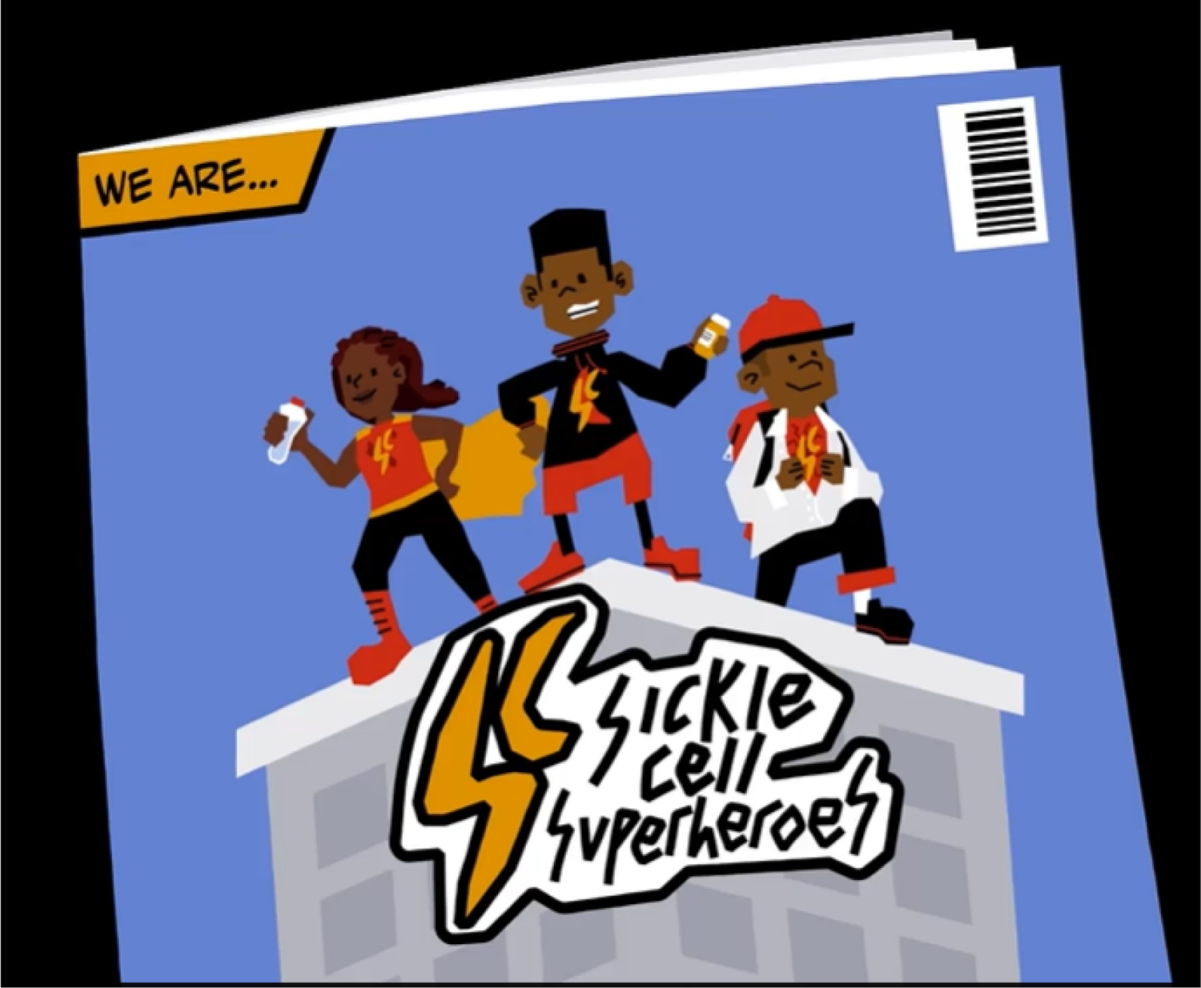
Sickle Cell Superheroes animation.

During the workshops, drafts of the script for the animation were shared with the CYP with SCD, their parents and researchers who edited it, so it was fit for purpose. Once it had been agreed, two of the CYP with SCD prepared the voiceover for the animation. The final animation (see Figure 8) was then reviewed and agreed upon before being published [21].

CYP involved in this project wrote the following about being involved in developing the project and the animation.

‘I really enjoyed [doing the voiceover] and I would love to do it again and I got to be a part of the project and have my voice heard. [It is important for researchers to work with CYP with SCD so] they can understand more from other people's point of view, especially children. So, the researchers know what we think as young people who have sickle cell. I learnt more about my sickle cell and how it works, and I learnt about what happens when the blood sickles in more detail. I learnt that there are other young people who have similar and different experiences to me.’

Edward, aged 10 years

‘I enjoyed sharing ideas for the animation and their superpowers. It is important that researchers work with children and young people with sickle cell disorder so that they can have more knowledge about sickle cell disorder and also share their own ideas as well. I gained a lot from being involved in the project and animation, I now have an experience on working with researchers as this was my first, learnt how information can be passed in different forms like animations, I also gained more knowledge about sickle cell disorder. For example, if people with sickle cell disorder are not properly hydrated, they will have crisis.’

David, aged 11 years

‘One of my favorite parts of working on the animation is how fast it went from our head, written down then magically added into the animation. I extremely loved everyone's ideas all so creative. I think it's very important to talk with children about Sickle Cell as then they themselves will be able to say, “because of my Sickle Cell sometimes my legs ache”. Something I will definitely take back from this animation process is that We have Sickle Cell, but Sickle Cell doesn't have us.’

James, aged 11 years

## 4 Discussion

Using a variety of different activities, we listened, heard and understood what is important for CYP with SCD and their families and used this information to co‐produce an animation [21] to enable inclusive and equitable PPIE with them.

CYP with SCD, their parents and researchers highlighted several considerations to ensure inclusive and equitable PPIE in research. While this study focused on CYP with SCD and their parents, both expressed the importance of including other family members, such as siblings, when designing or conducting research. It was acknowledged that long‐term health conditions can impact other members of the family aside from the affected child. This perceived ‘ripple effect’ is encompassed by Family Systems Theory (FST) [22]. FST focuses on the relationships between family members, including between parents, between the parents and child and other family members because they will all influence how the family functions. To understand the family and the way it functions, it is necessary to consider all family members and how they relate to each other, as well as their responsiveness to external influences [23] cited in [24]. In FST all components of the family are regarded as interdependent. What happens to one member will affect all other members of the family directly and indirectly [25, 26]. Therefore, when designing and conducting research with children with long‐term health conditions such as SCD, it is vital to consider the impact on the family in its entirety.

When designing or conducting research with CYP with SCD and their families, it is also important to consider the impact of stigma. Stigma has long been recognised as a potential barrier to access to healthcare for people with SCD. A recent integrative review found that negative attitudes, lack of knowledge and understanding of SCD, overt racism and racism due to implicit biases of healthcare providers led to disparities in care [27]. Although this review focused on the United States of America, a recent report in the United Kingdom also highlighted that people with SCD often receive sub‐standard care due to negative attitudes and racism [14]. A recent study found that similarly, attitudinal barriers, lack of awareness and racism can lead to mistrust which can hinder participation, engagement and involvement of ethnic groups in health research [7]. However, it is widely acknowledged that research should include those who gain to benefit the most. Therefore, strategies to involve people in research with conditions which primarily affect people from underserved and underrepresented, including CYP and minority ethnic groups, need careful consideration to overcome these barriers.

A major consideration in the current work was the practicalities of specifically working with CYP with long‐term health conditions. Previous guidance has suggested that consideration should be given to potential vulnerability, issues of capacity, legal protections when involving CYP in research and associated risks and benefits [6]. CYP, in the present study, expressed an interest in learning more about their condition. The work was not aimed at determining CYP's existing knowledge about SCD, and therefore, this was not assessed before their involvement. However, previous work has suggested that involving CYP in research may help develop emotional skills in terms of understanding that their views matter which can positively affect health outcomes [28]. Feedback from the CYP involved would appear to support this notion that involvement both inadvertently increased knowledge but also led to a more positive view of their condition and the impact on them. This study also highlighted many practical considerations that need to be considered, including flexibility around social clubs and activities children are already involved in, consideration of different attention spans according to the age and health condition of the child and their preferences in terms of morning, afternoon or evening working. Finally, making sure the research is not structured in such a manner that it is viewed as being an extension of an educational environment; although ground rules and consequences are important, undertaking preparatory work was viewed with the same disdain as homework by the CYP involved.

Careful consideration also needs to be given to the age and stage of development of the child. This is both in terms of how this enabled them to interact or not with children the same age, younger or older children and strategies to engage them. Previous work has indicated that adolescents with family heroes and adult peers as mentors (rather than celebrities/public figure heroes and same‐age peer mentors) demonstrated safer behaviours (*p* < 0.03) and higher interests in education (*p* < 0.03) [29]. However, the impact of adolescents as role models for younger children is less clear, but parents and children in the current study felt this could be a positive influence.

CYP, in the current study, stated from the outset that making the work fun and engaging was vitally important to sustain their interest. The Department for Education discusses the importance of using age‐appropriate strategies when working with CYP [30]. However, the challenge when undertaking research is finding activities that will appeal to children who span a range of age and development stages. The current study used a range of different online platforms to maintain the engagement of the CYP involved in the project over a period of 5 months of data collection.

The children in the present study expressed that they had enjoyed taking part in the research activities and found them informative and supportive. Using similar methods to engage children could be used in future to develop health initiatives to highlight the importance of engagement with health services and/or compliance with medication, as this has been shown to be problematic in people with SCD [31], particularly during the transition to adult care [32].

The current study had several limitations. It is recognised that recruitment to studies involving people with SCD can be challenging due to distrust of research, challenging life situations, debilitating chronic pain, stigma and logistic challenges such as transportation [15, 33]. Recruitment to the present study proved to be challenging, and therefore, the sample size is small, although in keeping with qualitative research. Attendance at workshops throughout the work was variable. This was primarily due to children being unwell and, therefore, both the parent and child being unavailable. The CYP included all who had SCD; therefore, the results may not be applicable to children with other health conditions. However, children ages ranged from 10 to 17 years, and they were recruited from several locations in England which may increase the transferability of the findings. There were a limited number of participants, but this enabled feasible moderation of the online environment used to conduct the project.

## 5 Conclusions

There are many potential barriers that need to be overcome to involve CYP with SCD and their families in research. These include the need for flexible timings, clear expectation setting, consideration of group dynamics and the impact of different ages and stages of development of the CYP involved, and ensuring appropriate recognition and compensation for their time. Listening, hearing and understanding what is important to CYP with long‐term conditions and using a variety of approaches is vital to support inclusive, equitable and sustained involvement and engagement in research.

## Author Contributions

Conceptualisation/study design: Jane Chudleigh. Data collection: Jane Chudleigh, Pru Holder, Addassa Follett, Ethan Mcfarlane‐Griffith, Derick Abuo, Josh Anon, and Imani Anon. Data analysis/data interpretation: Jane Chudleigh, Pru Holder, Addassa Follett, Ethan Mcfarlane‐Griffith, Derick Abuo, Josh Anon, and Imani Anon. Writing – original draft preparation: Jane Chudleigh and Pru Holder. Writing – review and editing: Jane Chudleigh, Pru Holder, Addassa Follett, Ethan Mcfarlane‐Griffith, Derick Abuo, Josh Anon, and Imani Anon. Visualisation: Jane Chudleigh and Pru Holder. Project administration: Jane Chudleigh, Pru Holder and Addassa Follett. Funding acquisition: Jane Chudleigh, Pru Holder and Addassa Follett. All authors have read and agreed to the published version of the manuscript.

## Ethics Statement

The study was conducted in accordance with the Declaration of Helsinki and approved by King's College London Research Ethics Committee Reference: LRM‐23/24‐38222.

## Consent

The Coalition for Personalised Care (C4PC) and NIHR have confirmed via email that permission is not required to use Figures 1 and 2, respectively. Written informed consent (and assent for children) was obtained from all subjects involved in the study.

## Conflicts of Interest

The authors declare no conflicts of interest.

## Data Availability

Data presented in this study are available on request from the corresponding author. Data are not publicly available due to ethical constraints.
